# DDtrek: A PyMOL-Based Management System for 3D Structural Data Series

**DOI:** 10.1021/acsomega.4c07417

**Published:** 2025-05-16

**Authors:** Evgenii M. Osipov, Sergei V. Strelkov

**Affiliations:** Laboratory for Biocrystallography, Department of Pharmaceutical and Pharmacological Sciences, 26657 Katholieke Universiteit Leuven, Herestraat 49, Box 822, 3000 Leuven, Belgium

## Abstract

In multiple areas of molecular biology research, series of closely related atomic structures of biomacromolecules are routinely generated. In particular, rational structure-based drug design heavily relies on the structural analysis of experimentally determined complexes between the target protein and multiple designed ligands (small molecules or biologicals). Here, we introduce DDtrek, a system for alignment, evaluation, and presentation of such structural data. This system is implemented as a plugin for the highly popular molecular graphics software PyMOL. A special feature of DDtrek is visualization of the aligned ligand structures together with the corresponding experimental density maps obtained through X-ray crystallography or cryoelectron microscopy, providing for the assessment of atomic model reliability and partial disorder. DDtrek is a lightweight and user-friendly system that can be utilized by whole research teams. DDtrek is freely available for Windows, MacOS, and Linux from https://github.com/BiocrystLab/DDtrek.

## Graphical abstract

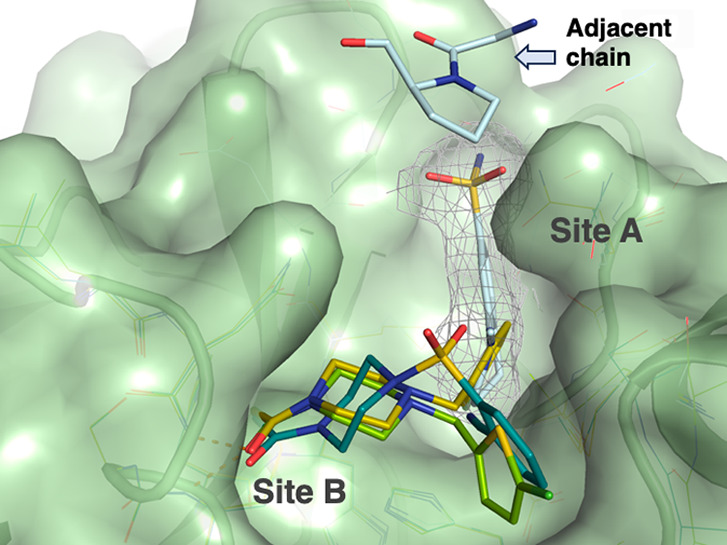

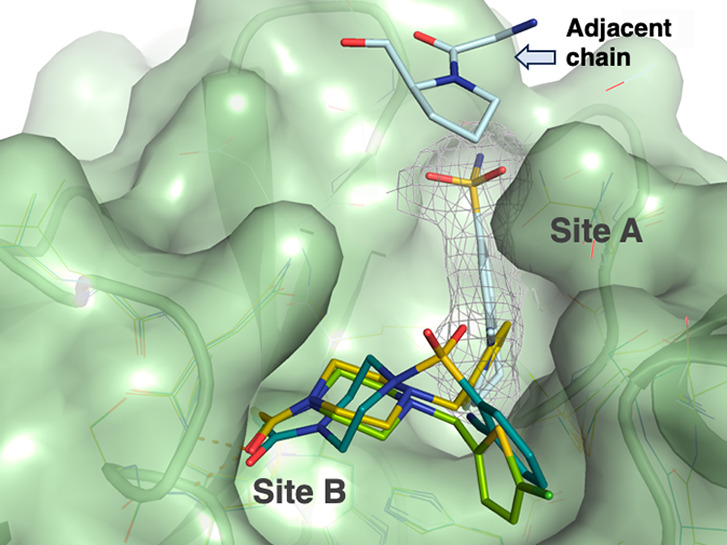

## Introduction

Three-dimensional structural data are an essential component of almost any scientific research involving biological macromolecules. Due to the continued advance of the three mainstream methods for atomic structure determination, i.e., the X-ray crystallography, nuclear magnetic resonance and cryogenic electron microscopy (cryoEM), such data are being generated at increasing speeds. Often, series of related experimental structures are being produced, such as, for instance, atomic structures of homologous proteins, multiple structures of the same protein in complex with different ligands or partner macromolecules, etc. In addition, high-quality structural data series for both individual proteins and macromolecular complexes can be generated through in silico prediction.

In particular, analysis of structural data series forms an important part of rational design of novel therapeutics.^1–3^ In general, such a design usually starts with some initial ‘hit’ compound with only modest affinity and activity toward the chosen macromolecular target. Next, the atomic structure of the target with the bound hit molecule needs to be determined. It is the analysis of three-dimensional structures through both human interaction and computational means which enables the downstream design of derivative compounds with improved characteristics. Most typically, this process is iterative, as multiple rounds involving structural studies, affinity/activity measurements and new design are necessary to arrive at a ‘lead’ compound with advanced characteristics. Along this path, hundreds to thousands of atomic structures are often generated.

Here, human inspection and analysis of the obtained structures using computer graphics is a key component. Numerous tools available for this purpose have a pedigree in structural biology such as Coot,^4^ Chimera,^5^ PyMOL,^6^ or CCP4MG,^7^ while others are part of in silico drug design packages, such as MOE,^8^ Schrödinger Small Molecule Suite/Maestro^9^ or ICM.^10^ Such tools typically include built-in algorithms for pairwise structural superposition, after which multiple structures can be analyzed side-by-side. Yet, for larger series of structural data, especially with experimental data, a customized solution is desirable which would enable automated reading of multiple coordinate files, their consistent structural alignment, and optimal organization in one visualization session. Our motivation here was to create a convenient tool to meet these needs, in particular, toward drug-design campaigns.

Here we describe DDtrek, a PyMOL plugin which enables easy comparative analysis of a series of structural data, such as complexes of a protein target with small-molecule ligands. We argue that PyMOL is particularly well suited for this purpose. It comes with an intuitive GUI providing access to basic functionalities, while built-in Python libraries enable powerful customization through scripting and custom functions. These factors contribute to a major popularity of PyMOL-based tools in structural biology. Importantly, through creating DDtrek, we hoped to meet the needs of ‘heterogeneous’ research teams i.e. those involving scientists with diverse expertise. In a typical scenario, DDtrek may be initially used by a structural biologist who feeds in the data including atomic coordinates but also, optionally, coefficients for rendering electron density maps (Figure 1A). Once the resulting PyMOL session file is shared with other team members, this session can be opened using a local PyMOL installation toward a specific interactive analysis. In particular, when multiple target-ligand complexes are analyzed, visualization of experimental maps provides direct assessment of the reliability of the ligand placement. In addition, further manipulations in PyMOL can be performed toward preparing publication-quality structural figures. While DDtrek is geared toward series of X-ray crystallographic structures of target/ligand complexes, it can also be used for other types of structure series and/or cryoEM maps. Additionally, DDtrek input file adds layer of data management and allows to follow FAIR principles of scientific data management.^11^

**Figure 1:**
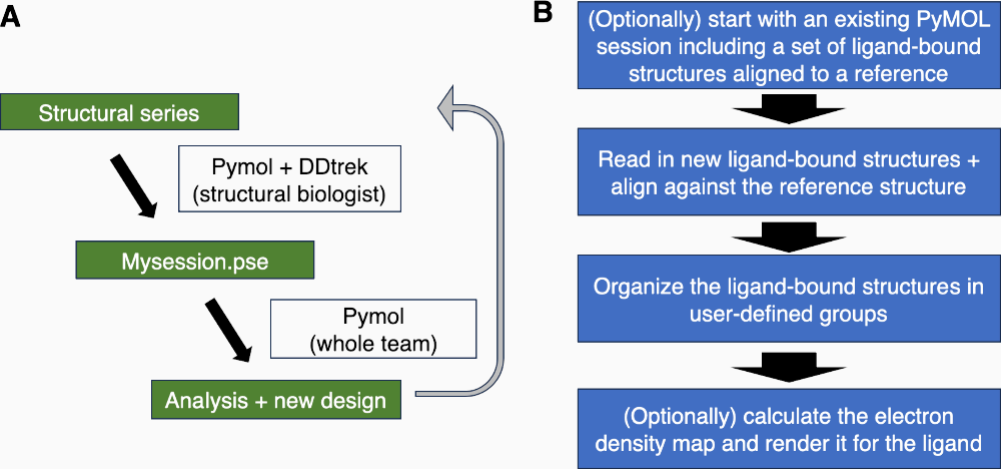
DDtrek plugin. (A) Schematic of intended use. (B) Overview of the algorithm.

## Results

### Installation

The DDtrek plugin is written in Python 3 and mostly uses the built-in PyMOL functions and libraries. The only external dependency is the Gemmi library.^12^ Specifically, coordinate manipulation is performed by PyMOL directly, while the Gemmi library handles operations with electron density maps. The plugin should be used with PyMOL version 2.5 or later, since older versions of PyMOL work unreliably with the Gemmi library. The source code of DDtrek and examples are freely available from https://github.com/BiocrystLab/DDtrek under the GPLv3 license. The plugin could be directly downloaded using the link https://raw.githubusercontent.com/BiocrystLab/DDtrek/main/ddtrek.zip

The DDtrek plugin is supplied as a single file ddtrek.zip and should be installed using the PyMOL plugin manager (Figure 2A). The plugin can be installed under Windows, MacOS and Linux. After installation, a graphical interface becomes available through the ‘Plugin’ menu. Graphical interface is used to load a formatted input file (Figure 2B).

**Figure 2:**
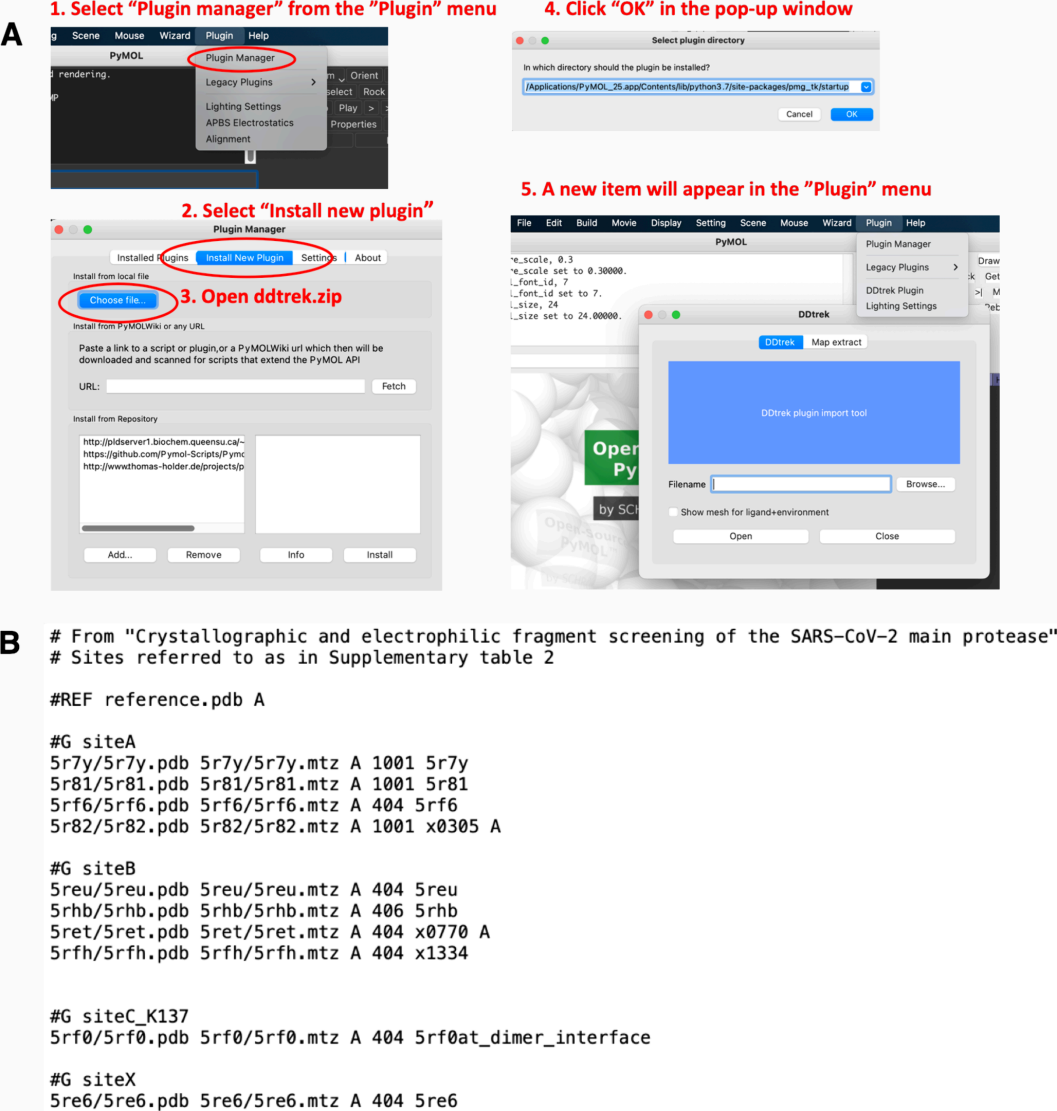
(A) DDtrek installation process. (B) Example of an input file.

### DDtrek Algorithm

On input, DDtrek requires a set of atomic structure files. One of these serves as a reference structure. Users can specify a particular protein structure as a refence. If reference is not specified, DDtrek will use the first structure specified in the input file. All other structures will be aligned to this reference (Figure 1B). If these structures include multiple chains, the user should specify the chains to be aligned.

After alignment, each structure is truncated around a particular residue specified by its chain and residue numbers. This could either be a protein residue or a bound ligand. For all or some structures, phased crystallographic data or cryoEM maps can also be provided. In these cases, DDtrek will truncate and render the density map around the specified residue.

### Implementation

Before using the plugin, one should either start a new PyMOL session or load a previously saved session. In the latter case, the session may already contain various structural objects created during the previous run of DDtrek, including ligand-bound structures and the reference structure. Next, the plugin is used to open a text file with input instructions. An example of such a file is given in Figure 2B.

First, DDtrek checks for the presence of a reference structure object (named *reference*) in the active PyMOL session. Unless this object is there already, the PDB file containing the reference may be specified in the first line of the input file after the tag #REF. If the reference structure was not loaded already or is also not specified explicitly, the first loaded structure will be used. The reference object always consists of a single protein chain even if the target protein is multimeric. To select a specific chain, the name of the input file should be followed by a chain identifier otherwise the first chain is selected.

The remainder of the input file specifies the structures to be aligned with the reference. By design, DDtrek arranges all rendered structural objects in groups, using the functionality available in PyMOL. The list of groups and objects will appear in the right-hand panel of the PyMOL GUI. The way the groups are organized is up to the user; this could be used e.g. for multiple ligands with a common backbone or ligands binding in the same site. To this end, the input card should contain at least one line with the tag #G <groupname> to specify a new group. This should be followed by the information on the structures to be included in this group. Further tags #G <groupname> initiate additional object groups.

Per structure, the following information needs to be provided on one line:

<PDB file with coordinates> <Optionally: MTZ file with map coefficients> <Chain ID of the ligand/residue of interest> <Residue range of the ligand> <Object name> <Optionally: protein chain ID for alignment with reference>

Location of the files can be specified either with an absolute or relative path. For each input structure, DDtrek first checks whether the object with the same displayed name is present already. If this is the case, the object is skipped. Otherwise, a new structural object is created. Ligands or residues of interest are uniquely identified using chain and residue identifiers. Here, syntax of the identifiers follows PyMOL notation, hence chain and residue identifiers could be specified using single number or range, e.g., 100 or 100–102. Next, DDtrek uses the crystallographic symmetry to add protein residues coming from symmetry-related chains in the vicinity of the specified ligand. Thereafter the object is structurally aligned with the reference. To this end, one chain of this structure (the first one or the one specified explicitly) is superimposed with the reference structure using the super function of PyMOL. After structural alignment, the new object is truncated to all protein residues (possibly coming from several protein chains in the asymmetric unit and/or symmetry mates) located within 4.5 Å of the ligand. Finally, carbon atoms of the object are rendered using a randomly selected color from the predefined PyMOL palette.

If an mtz file with map coefficients is provided in the input file, DDtrek will extract a fragment of the electron density map within 3 Å from the specified ligand. The current version only supports extraction of weighted 2Fo-Fc maps specified by the coefficients labeled FWT, PHWT (as output by the program Refmac5) or 2FOFCWT, PH2FOFCWT (as output by Phenix_refine or Buster) in the mtz file. As an alternative to using MTZ files, DDTrek can directly read density maps (specified as the second parameter per line of the input file, see above), including cryoEM maps or crystallographic Polder difference maps.^13^ The map is translated and rotated using the transformation matrix produced during the alignment of the ligand-bound structure to the reference structure. By default, DDtrek generates a new mesh representation for the map at 1σ level carved within 1.8 Å from the ligand atoms and stores it in the object named *<structural_object_name>_mesh*. If the GUI option “Show mesh for ligand+environment” is additionally selected, DDtrek will draw the mesh not only for the ligand but also for all protein residues located within extracted map fragment. Finally, the mesh is assigned the same color as the main color of the ligand. Density maps are stored in a separate group called *map_objects*. They should be retained to preserve the possibility of redrawing meshes at different levels or with a different color.

As the last step, the user should save the PyMOL session with a desired name. Since the input structures and density maps are trimmed around the ligand, the resulting session file size is small (about 0.1 MB per structure including a density map). The session size could be reduced further by discarding the map objects or saving the session in a compressed (pze) format. Of note, the resulting PyMOL session can be viewed using a range of PyMOL versions, including older ones, and on all major operating systems.

The second tab of DDtrek GUI “Map extract” is a standalone function for the extraction of a map fragment around the specified selection. This tab can be used to extract a fragment from a cryoEM or a crystallographic density map. To reduce the size of the resulting Pymol session, DDtrek extracts a smaller fragment of the map within 9 Å from the relevant selection. By default, the map fragment is saved into the ‘extracted_map’ object, which could be further visualized according to the needs.

### Case Study

To illustrate the application of DDtrek, we utilized the structural data from the COVID Moonshot project, an open-science drug discovery campaign targeting the main protease (MPro) of SARS-CoV2.^14^ Here, direct crystallography-based screening with a library of drug-like fragments resulted in the identification of 74 binders, including 48 covalent and 23 noncovalent hits. Using DDtrek, we rendered selected bound fragments while grouping them according to the subpocket they occupy (site A or site B, Figure 3). Of note, DDtrek detected the presence of a symmetry-related protein chain (i.e., a crystal lattice contact) in the vicinity of site A, as shown in the figure. On the one hand, this may prevent the binding of more bulky fragments. On the other hand, the binding of smaller fragments could be additionally stabilized in the crystal relative to solution, and thus artifactual.

**Figure 3:**
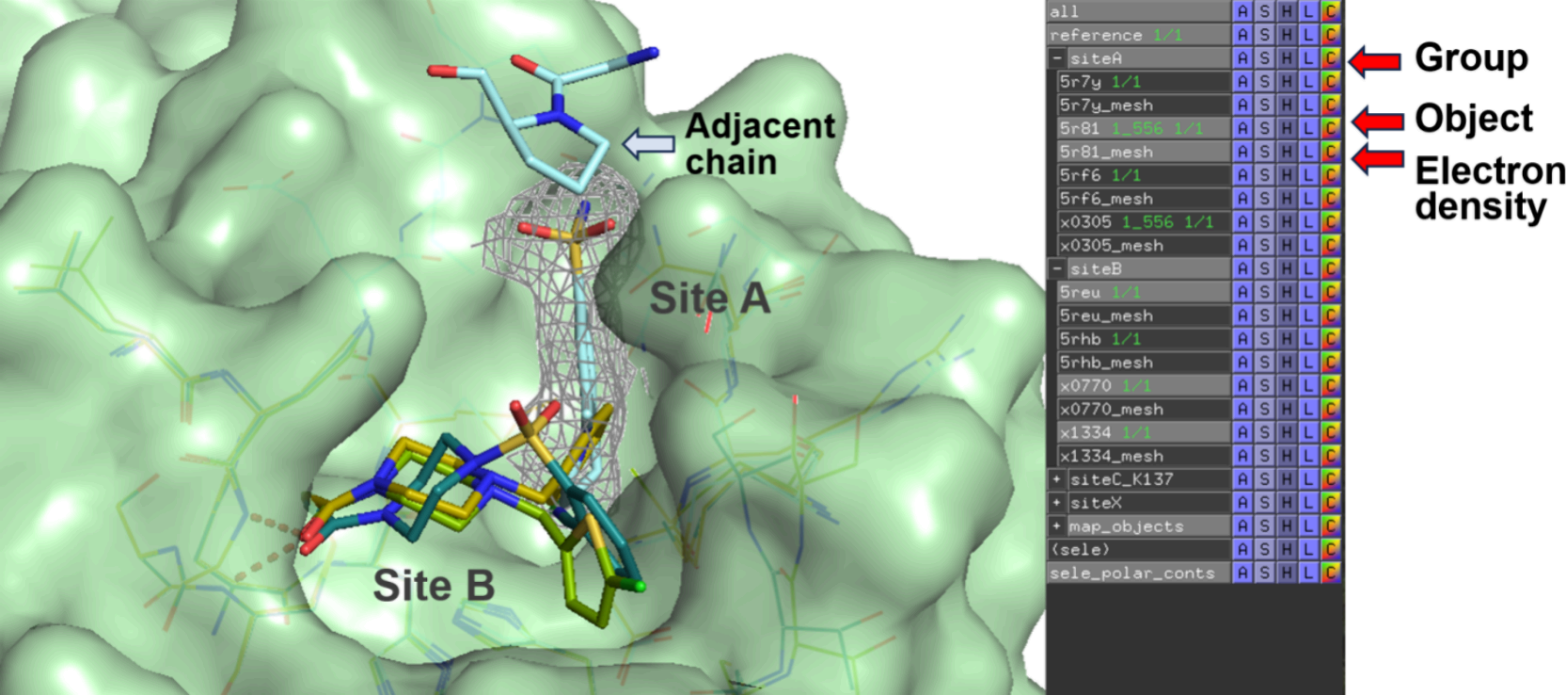
Screenshot of the PyMOL session for a series of structures obtained during a fragment screening campaign against the main protease of SARS-CoV2.^14^ The figure was generated using DDtrek with the input file shown in Figure 2. One fragment in site A (blue sticks) is rendered together with its electron density (gray mesh). Residues of a close symmetry-related protein chain are also shown. In addition, several overlapping fragments binding in site B are also drawn. These fragments make H-bonds to residues in the cavity (orange dashed lines).

Another example (see https://github.com/BiocrystLab/DDtrek/tree/main/examples/Paur_FabF) comes from a fragment screening campaign with β-ketoacyl-[acyl-carrier-protein] synthase II (FabF) from Pseudomonas aeruginosa.^15^ Crystals of the fragment-bound FabF belong to two distinct crystal forms (exemplified by PDB entries 5sno and 5snp), meaning that structural alignment is necessary prior to comparing the ligand poses.

## Discussion

DDtrek was conceived as a user-friendly and portable solution to store, visualize and analyze series of 3D structural data on biological macromolecules, such as drug-target complexes in particular. While pairwise alignment of atomic coordinates is a standard routine available through various molecular graphics software, our purpose was to enable automated and convenient comparison and analysis across larger series of structures.

To this end, we consciously chose PyMOL as the graphic engine. This choice brings multiple benefits. PyMOL is very user-friendly and extremely popular, meaning that a vast number of researchers in the field are already routinely using this software. Once the individual structures are added and aligned via DDtrek, they will appear as individual objects in the right-hand window of the PyMOL GUI and can additionally be organized in groups. Through pressing Ctrl-F one can activate a search by object or group name. Objects and groups can be reorganized further through dragging with a mouse. Moreover, the structures can be rendered and analyzed in various ways thanks to the rich functionality of PyMOL. For instance, the binding cavity may be drawn as a semitransparent surface, and various types of ligand interactions can be detected using the Action > Find pull-down menu. Last but not least, the use of PyMOL enables the generation of highly customizable, high-quality figures for presentation and publication purposes.

An important asset of DDtrek is the possibility to render the aligned structures together with experimentally determined density maps. This reveals the reliability of model building for the relevant part (such as the bound ligand) that had been done by the scientist upon structure solution. Even if there are no explicit mistakes, the spatial resolution of the map is often too low, or the map is too noisy for the ligand to be placed reliably. If necessary, DDtrek can input a mix of experimental structures (with maps) and in silico produced structures such as predicted ligand complexes, enabling their direct comparison. Moreover, we often see cases when the quality of the density map varies across the different groups comprising the ligand. Typically, this happens because certain parts of the ligand remain mobile in the bound state. Such observations are invaluable, in particular, when making informed decisions on ligand optimization. Figure 4 shows an example from our own recent rational design project targeting the PWWP domain of the HRP family.^16^ Here, the parent compound **41** reveals a very good density when bound to the target (Figure 4 A). The derivative compound **42** has a 4-aminotoluene extension, but the latter is only poorly resolved in the map (Figure 4 B). This extension is involved in a rather weak interaction with the target cavity via a water bridge. In line with this, the derivative compound shows only a minor 1.5x improvement in the affinity value compared to the parent compound.

**Figure 4:**
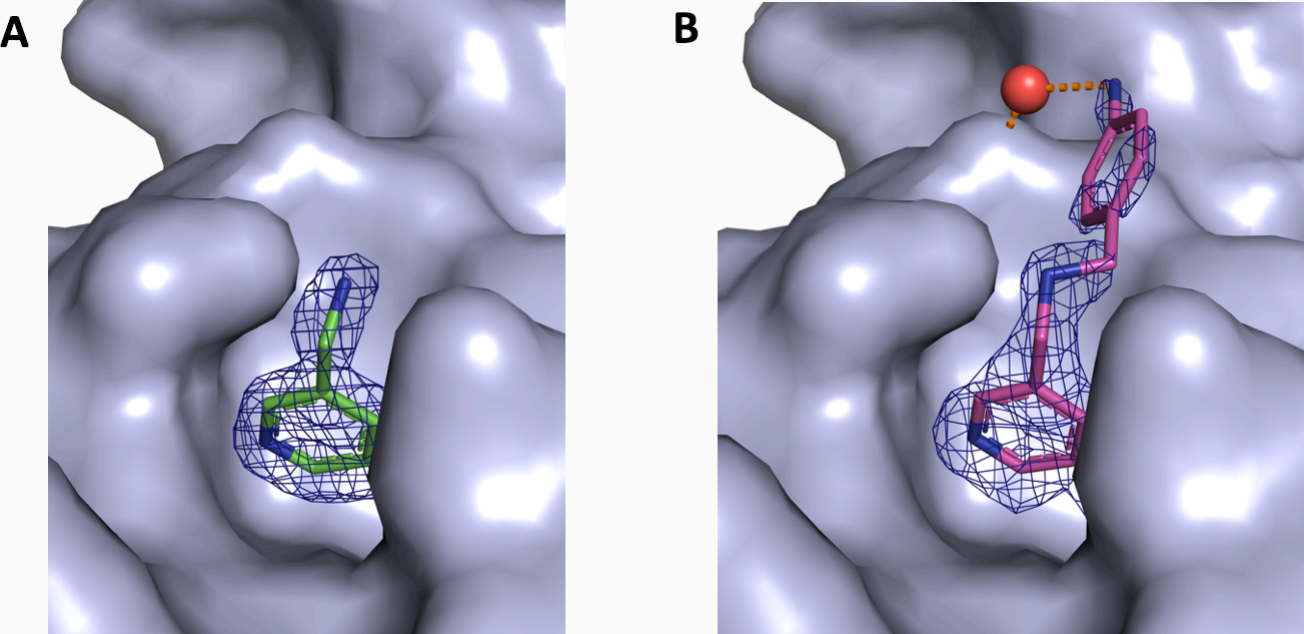
Importance of analyzing density maps, as exemplified by data from Vantieghem et al.^16^ (A) Parent compound **41** rendered together with its 2Fo-Fc electron density map at 1.34 Å resolution and 1σ level. (B) The derivative compound **42** (PDB entry 9G96) carrying a 4-aminotoluene extension together with its electron density at 1.8 Å resolution and 1σ level. An additional H-bond to a structured water molecule (red sphere) is shown with an orange dashed line.

Of note, a server-based tool called Fragalysis is currently being developed at the XChem facility of the Diamond synchrotron (UK) toward the analysis of structural fragment screening data.^17,18^ The main functional capacities of DDtrek and Fragalysis are similar. However, while Fragalysis was designed to integrate into the XChem fragment screening pipeline,^19^ DDtrek can be readily run using a standard PyMOL instance. It should also be noted that DDtrek (just like Fragalysis in the current implementation) is less suitable toward interactive building/modification of the ligands. Even though PyMOL does provide some functionality in this direction, advanced in silico design packages (MOE, Schrödinger Small Molecule Suite, SeeSAR^20^ etc.) are better suited here. These packages include additional utilities which aid in this process, such as local energy minimization and stereochemical restraints.

## Data Availability

Code and examples are available from https://github.com/BiocrystLab/DDtrek/.
